# Transposed-word effects when reading serially

**DOI:** 10.1371/journal.pone.0277116

**Published:** 2022-11-10

**Authors:** Jonathan Mirault, Aaron Vandendaele, Felipe Pegado, Jonathan Grainger

**Affiliations:** 1 Laboratoire de Psychologie Cognitive, Centre National de la Recherche Scientifique & Aix-Marseille University, Marseille, France; 2 Pôle pilote Ampiric, Institut National Supérieur du Professorat et de l’Éducation, Aix-Marseille Université, Marseille, France; 3 Department of Experimental Psychology, Ghent University, Ghent, Belgium; 4 Institute of Language, Communication and the Brain, Aix-Marseille University, Marseille, France; Max Planck Institute for the Science of Human History, Jena, Germany, HUNGARY

## Abstract

When asked to decide if an ungrammatical sequence of words is grammatically correct or not readers find it more difficult to do so (longer response times (RTs) and more errors) if the ungrammatical sequence is created by transposing two words from a correct sentence (e.g., *the white was cat big*) compared with a set of matched ungrammatical sequences for which transposing any two words could not produce a correct sentence (e.g., *the white was cat slowly*). Here, we provide a further exploration of transposed-word effects while imposing serial reading by using rapid serial visual presentation (RSVP) in Experiments 1 (respond at the end of the sequence) and 2 (respond as soon as possible—which could be during the sequence). Crucially, in Experiment 3 we compared performance under serial RSVP conditions with parallel presentation of the same stimuli for the same total duration and with the same group of participants. We found robust transposed-word effects in the RSVP conditions tested in all experiments, but only in error rates and not in RTs. This contrasts with the effects found in both errors and RTs in our prior work using parallel presentation, as well as the parallel presentation conditions tested in Experiment 3. We provide a tentative account of why, under conditions that impose a serial word-by-word reading strategy, transposed-word effects are only seen in error rates and not in RTs.

## Introduction

The present study provides a further investigation of transposed-word effects obtained with the grammatical decision task and first reported by Mirault et al. [1]. Mirault et al. presented participants with a mixture of grammatically correct 5-word sentences (e.g., *The car was light blue*) and ungrammatical 5-word sequences, and asked them to respond as rapidly and accurately as possible if the sequence of words they read was grammatically correct or not. The key manipulation in the Mirault et al. [1] study concerned the nature of the ungrammatical sequences, which could be formed by transposing two words in a correct sentence (e.g., *The white was cat big*), and control sequences (e.g., *The white was cat slowly*) for which a grammatically correct sentence could not be formed by transposing any two words. Mirault et al. found that the transposed-word sequences were harder to classify as being ungrammatical compared with the control sequences—a transposed-word effect (see Liu et al. [2]; Liu et al. [3], for a replication of transposed-word effects in Chinese). In a follow-up study, Snell and Grainger [4] showed that transposed-word effects were greater when the transposition involved two adjacent internal words (e.g., *the was man old*) compared with non-adjacent external word transpositions (e.g., *old man was the*). Snell and Grainger argued that these findings were evidence for the noisy bottom-up assignment of word identities to their positions in a line of text as one key source of transposed-word effects.

Transposed-word effects have also been reported in the psycholinguistic literature (principally with auditory stimuli and using off-line techniques such as non-speeded grammaticality or well-formedness judgments) where it has been demonstrated that given the goal to understand / interpret linguistic input, adult humans are capable of recovering from various types of error including errors in word order (see Mollica et al. [5], for a review). One influential account of how readers can recover from such errors is the noisy channel model proposed by Gibson, Levy, and colleagues (e.g., Gibson et al. [6]; Levy et al. [7]; Ryskin et al. [8]). The general idea is that noisy input to sentence-level processing provides support for what has been referred to as “good-enough” representations for language comprehension (e.g., Ferreira & Lowder [9]; see also the “lossy-context surprisal” model of Futrell et al. [10]). Such good-enough or incomplete representations of the input would promote sentence interpretability in the face of ungrammaticality caused, for example, by changes in word order. Thus, noisy incremental models of language comprehension can account for transposed-word effects, and therefore such effects are not necessarily a signature of parallel word processing (Huang & Staub [11–13]; Milledge et al. [14]). Support for this general approach was provided by Dufour et al. [15] who found robust transposed-word effects with an auditory grammatical decision version of the Mirault et al. [1] study. Given that speech imposes serial word processing, this implies that transposed-word effects can be observed under strictly serial processing conditions. In the present work we provide a further investigation of transposed-word effects when imposing serial processing in the visual modality.

**Fig 1:**
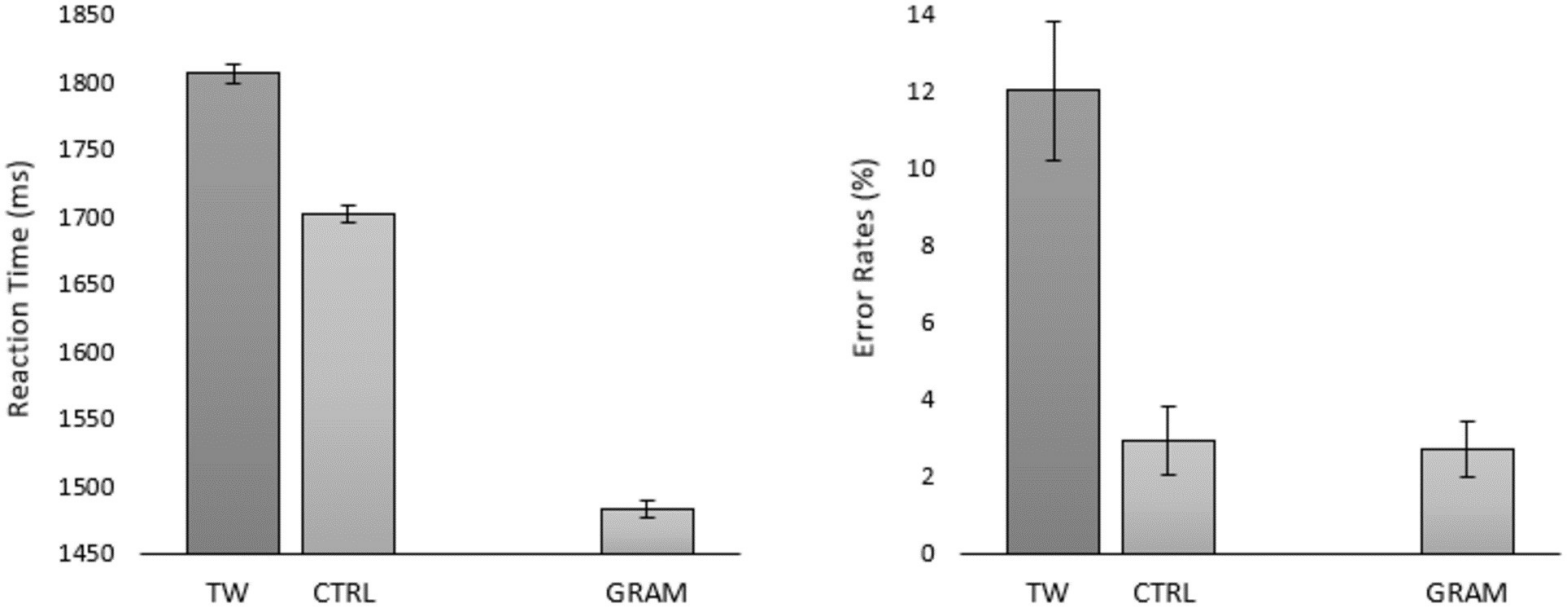
Results of the on-line experiment reported in Mirault et al. (2018) for transposed-word (TW) and the corresponding control (CTRL) ungrammatical sequences and the correct sentences (GRAM). Error bars are 95% confidence intervals (Cousineau [16]).

To do so, we investigated transposed-word effects under serial, one-word-at-time, presentation conditions using the Rapid Serial Visual Presentation (RSVP) procedure. All experiments tested for transposed-word effects using the same set of 5-word sequences and the grammatical decision task as in Mirault et al. [1]. Given that we used the materials of Mirault et al. [1] under on-line testing conditions, we first remind readers of the results of the on-line study published in the Mirault et al. paper. The key point to note here is that transposed-word effects (i.e., the difference between the transposed-word and control conditions) were significant in both response times (RTs) and error rates (see Fig 1). Further note that the results of the on-line study aligned perfectly with the laboratory study reported in the same paper.

One highly relevant prior study has investigated transposed-word effects under both parallel and serial presentation conditions in the visual modality in Chinese (Liu et al. [17]). This study reported significant transposed-word effects in both RTs and error rates under parallel presentation, and a significant effect under serial presentation, but only in the error rates. Moreover, the transposed-word effect in error rates was significantly greater under parallel presentation. The present work provides an extension of this prior study by investigating transposed-word effects under visual serial presentation conditions in French (in order to compare the results with those obtained by Mirault et al. [1]), and by providing a direct comparison of the effects obtained under serial and parallel presentation conditions. Following Liu et al. [17], in Experiments 1 and 2 we imposed serial reading by using the RSVP procedure with words presented briefly one after the other on the center of the screen. In Experiment 1 participants could only respond at the end of the RSVP stream, while in Experiment 2 they could respond at any time (i.e., before the end of the sequence if possible—see also Huang & Staub [13]). The latter procedure allowed us to have RTs in the transposed-word condition roughly in line with those obtained in the Mirault et al. study. Crucially, in Experiment 3 we provide a direct comparison of transposed-word effects obtained with the parallel presentation of word sequences and serial presentation using the RSVP procedure. Our overarching hypothesis was that conditions that impose serial word processing should reduce bottom-up word position uncertainty, hence reducing transposed-word effects compared with the effects obtained under parallel word presentation (Mirault et al. [1]).

## Experiment 1

### Methods

#### Participants

Two hundred and eight participants (82 female) took part in the experiment. They all were native speakers of French, ranged in age from 18 to 72 years (M = 29, SD = 9.85), and received monetary compensation (£9/hour). They were naïve to the purpose of the experiment and signed online an informed consent form before starting the experiment. The large number of participants was designed to provide a more robust investigation of the null effect of word transposition seen in the RT data obtained in pilot studies. The large number of participants also enabled a stronger test of the null effect in RTs by randomly sampling a sub-group (N = 50) of participants in 5 independent follow-up analyses.

#### Design & stimuli

We applied the design of Mirault et al. [1], in which a grammatical decision task was used to compare two critical conditions: the transposed-word condition (i.e.; an ungrammatical sequence of words formed by transposing two words in a correct base sentence) and the control condition (i.e., a sequence composed of all but one word of the transposed-word sequence, but where transposing any two words could not generate a correct sentence). In order to be able to compare the present findings with the original Mirault et al. [1] study we used the same 160 five-word French sequences (80 grammatically correct, 40 ungrammatical transposed and 40 ungrammatical controls) tested by Mirault et al. [1] (see the original paper for more details of the stimuli). As in the Mirault et al. study a Latin-square design was used such that each version of an ungrammatical sequence (i.e., the transposed-word version vs. control version) was seen an equal number of times across participants, but only once per participant. Thus, half of the participants saw one version of the transposed-word / control sequence pairs (see Table 1) in one condition (e.g., “the white was cat big”) and the other half in the other condition (e.g., “the white was cat slowly). The correct sentences served as fillers to meet the task requirement and were excluded during the statistical analyses. English examples of each condition can be found in Table 1.

**Table 1:** Examples of stimuli used in the experiments.

*Condition*	French example	English example
Base sentences	Ton petit chat avait faim	The white cat was big
	Cette grande tasse est cassée	The black dog ran slowly
Transposed-word sequences	Ton petit avait chat faim	The white was cat big
	Cette grande est tasse cassée	The black ran dog slowly
Control sequences	Ton petit avait chat cassée	The white was cat slowly
	Cette grande est tasse faim	The black ran dog big

Note. Examples are also provided in English for convenience (they are not translations of the French examples). The critical ungrammatical sequences were constructed from pairs of base sentences. The correct sentences in the experiment, included for the purpose of the grammatical decision task, were not the same as the base sentences (i.e., participants were not shown the base sentences).

#### Procedure

**Fig 2:**
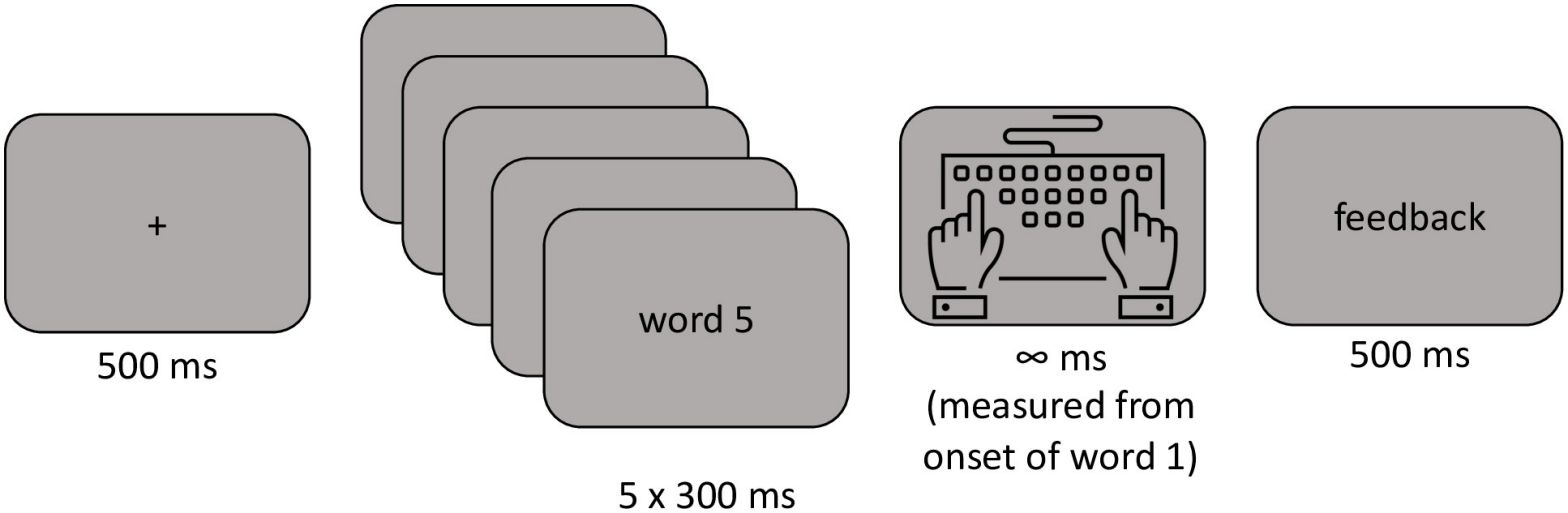
Schematic of the RSVP procedure used in Experiment 1. Participants saw a sequence of 5 five words each for 300 ms and centered on the screen and could only respond when the last word disappeared. RTs were measured from onset of word 1 in order to be compatible with the other experiments. Participants responded with the left and right arrow keys on their computer keyboard.

The experiment was created using LabVanced (Finger et al. [18]) and we used the Prolific platform (Palan & Schitter [19]) to recruit participants online. Each trial started with a fixation cross displayed in the middle of the screen during 500 ms. Then, a 5-word sequence was rapidly presented word-by-word, with each word staying onscreen for 300 ms (see Fig 2). Participants were instructed to respond at the end of the word sequence and RTs were measured from onset of the first word (recording from onset of the final word would, of course, give identical results with shorter RTs). Participants were requested to judge as rapidly and as accurately as possible if the sequence of words formed a grammatically correct sentence or not. Participants responded using the right and left arrow keys on their computer keyboard (right for a “yes” response, left for a “no” response). Next, feedback was given during 500 ms: a green dot for a correct response and a red cross for an error. Providing feedback ensured that there was no ambiguity as to what constituted a grammatically correct sentence. Participants received 6 practice trials for familiarization. The order of trials was randomized for each participant.

#### Analyses

Both RTs and error rates were analysed separately as dependent variables. This was done by using linear mixed-effects (LME) and generalized logistic mixed-effects (GLME) models respectively, fitted in the RStudio version 3.6.1 (R Core Team [20]) statistical computing environment using the *lme4* package (Bates et al. [21]). In each model, Transposition (transposed-word vs. control) was a fixed effect, whilst including the by-item and by-participant factors, as well as their interaction as crossed random effects where possible (Baayen et al. [22]). This resulted in the reporting of *b*-values, standard errors (*SEs*) and *t*- or *z*-values (for RTs and error rates respectively), with values beyond |1.96| deemed as significant (Baayen [23]). A logarithmic transformation (log_10_(RT)) was performed to normalize the RT data. Statistical analyses were only performed on the data obtained with the two types of ungrammatical sequences. Results for the correct sentences are provided for comparison. All the models, in this experiment and the following experiments, included random intercepts and random slopes whenever possible. We also provide an analysis of conditional accuracy functions to examine the extent to which effects in error rates vary as a function of average RT (Bonnet & Dresp [24]; Fernández-López et al. [25]). These analyses provide crucial additional information given the importance of the effects obtained in error rates in the present study.

### Results

**Fig 3:**
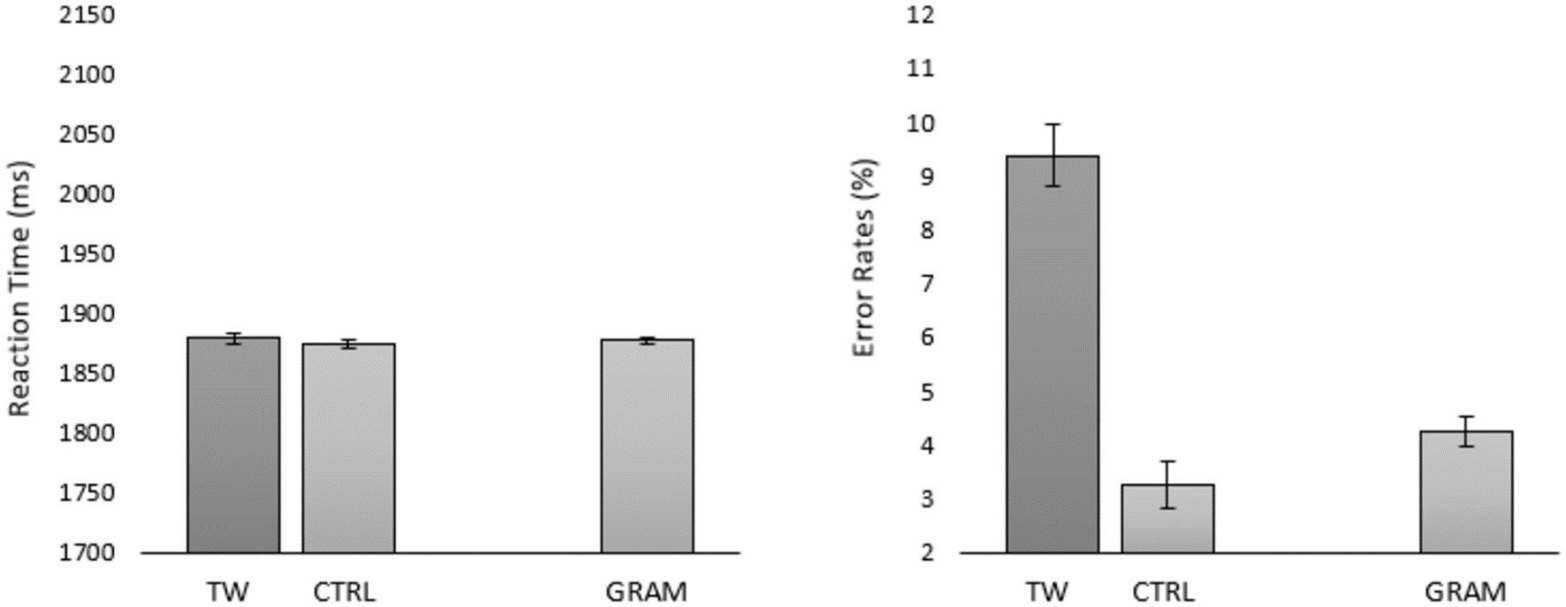
Mean RTs and error rates per condition in Experiment 1. Error bars represent within-participant 95% CI (Cousineau [16]). *TW* = transposed-word, *CTRL* = control, *GRAM* = grammatically correct.

Prior to statistical analysis, we excluded 6 participants with accuracy lower than 75%. We also excluded trials with RTs < 100 ms and > 3,000 ms (4.93%). The remaining dataset was composed of 31,028 observations. In this and the following experiments the number of data points per condition largely exceeded the number recommended by Brysbaert and Stevens [26] for sufficient power. Condition means for RTs and error rates are shown in Fig 3. The results for the grammatically correct sentences are shown for comparison. Raw data of all experiments are available on OSF: https://osf.io/raxgj/.

#### Response times

Prior to analysis, we removed incorrect responses (5.21%) and trials with values beyond +/- 2.5 SD from the grand mean (2.92%). The effect of Transposition was not significant (*b* = 1.16; SE = 0.75; *t* = 1.53), with practically equivalent RTs being obtained to the transposed-word sequences and the control ungrammatical sequences.

#### Error rates

There was a significant effect of Transposition (*b* = 1.28; SE = 0.18; *z* = **6.89**) with more errors being made to the transposed-word sequences compared with the control sequences.

#### Conditional accuracy functions

**Fig 4:**
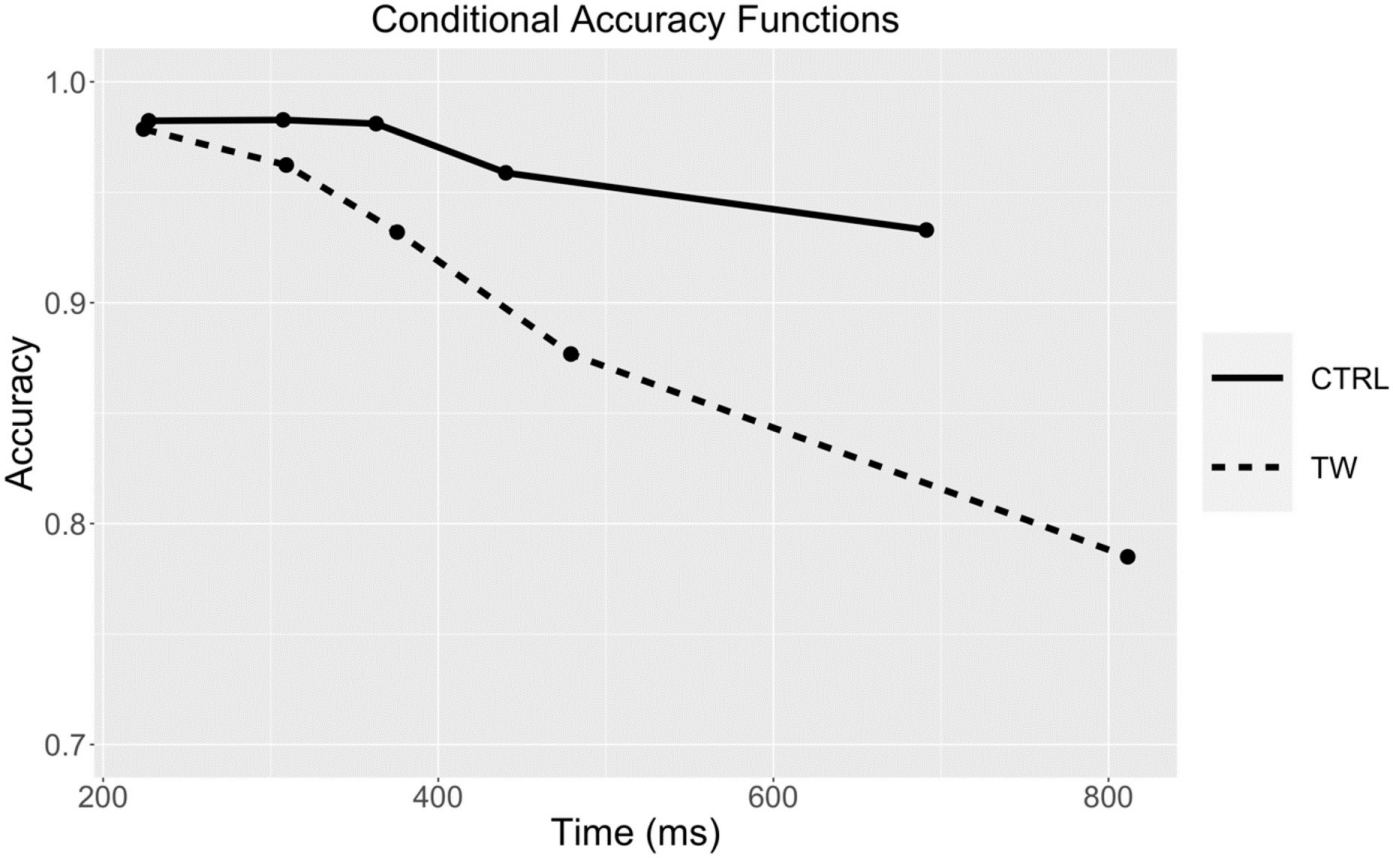
Conditional accuracy functions for the Transposed-word (TW) and Control (CTRL) conditions in Experiment 1. The points represent the accuracy (probability correct) and average RT (in ms) of responses within equal-sized bins (20% of responses per bin).

Fig 4 shows the conditional accuracy functions for the two types of ungrammatical sequence obtained with the RSVP procedure used in Experiment 1. These functions plot the evolution of accuracy with changes in average RT measured in equal sized bins (here 20% bins—i.e., the 20% fastest RTs, then the next fastest 20%, etc.). The division of RTs into bins involves both correct and incorrect responses, such that accuracy can be calculated for each bin.

#### Test of robustness of effects

Given the theoretical importance of the pattern of effects observed in Experiment 1 (transposed-word effects in error rates by not in RTs) and given the large sample of participants that were tested, the robustness of this pattern was put to test by randomly sampling 50 participants and re-analyzing the data associated with these 5 different random re-samplings. After randomly selecting 50 participants for each analysis (25 participants from each of the two counterbalanced groups), we applied the same analysis procedure as for the main analysis. Outputs of LMEs and GLMEs are reported in Table 2.

**Table 2:** Outputs of LMEs (RT) and GLMEs (error rate) for 5 random samplings of 50 participants in Experiment 1.

Sample	Response time			Error rate		
	*b*	SE	*t*	*b*	SE	*z*
N°1	1.95	1.29	1.50	1.17	0.30	**5.66**
N°2	2.06	1.27	1.63	1.30	0.15	**8.7**
N°3	0.22	1.04	0.21	1.13	0.36	**3.11**
N°4	0.84	1.28	0.65	1.55	0.24	**6.33**
N°5	0.26	1.25	0.21	1.23	0.33	**3.75**

### Discussion

The results of Experiment 1 revealed significant transposed-word effects in error rates but not in RTs. The robustness of this pattern was demonstrated using a random sampling of participants, which was made possible by the large number of participants that were tested in this experiment. We found the same pattern when randomly sampling 50 participants in 5 independent follow-up analyses. Moreover, the conditional accuracy functions revealed that the effect in error rates increased as average RT increased (see Fig 4). However, the fact that there was no evidence for a transposed-word effect in RTs in Experiment 1 could be because participants could only respond at the end of the sequence of words. In order to test whether a transposed-word effect would emerge in RTs when participants could respond at any time during the presentation of the word sequence, we ran an additional RSVP experiment.

## Experiment 2

### Methods

#### Participants

One hundred and one participants (42 female) took part in the experiment. They all were native French speakers ranging in age from 18 to 71 years (M = 28.04, SD = 9.01), and received monetary compensation (£9/hour). They were naïve to the purpose of the experiment and signed online an informed consent form before starting the experiment.

#### Design & stimuli

The same as Experiment 1.

#### Procedure

The procedure was the same as in Experiment 1 except that now participants could respond before the end of the 5-word sequence, and the RSVP stream stopped after participants’ response (i.e., with a response during the presentation of word 3, words 4 and 5 were not presented).

#### Analyses

The same as for Experiment 1.

### Results

**Fig 5:**
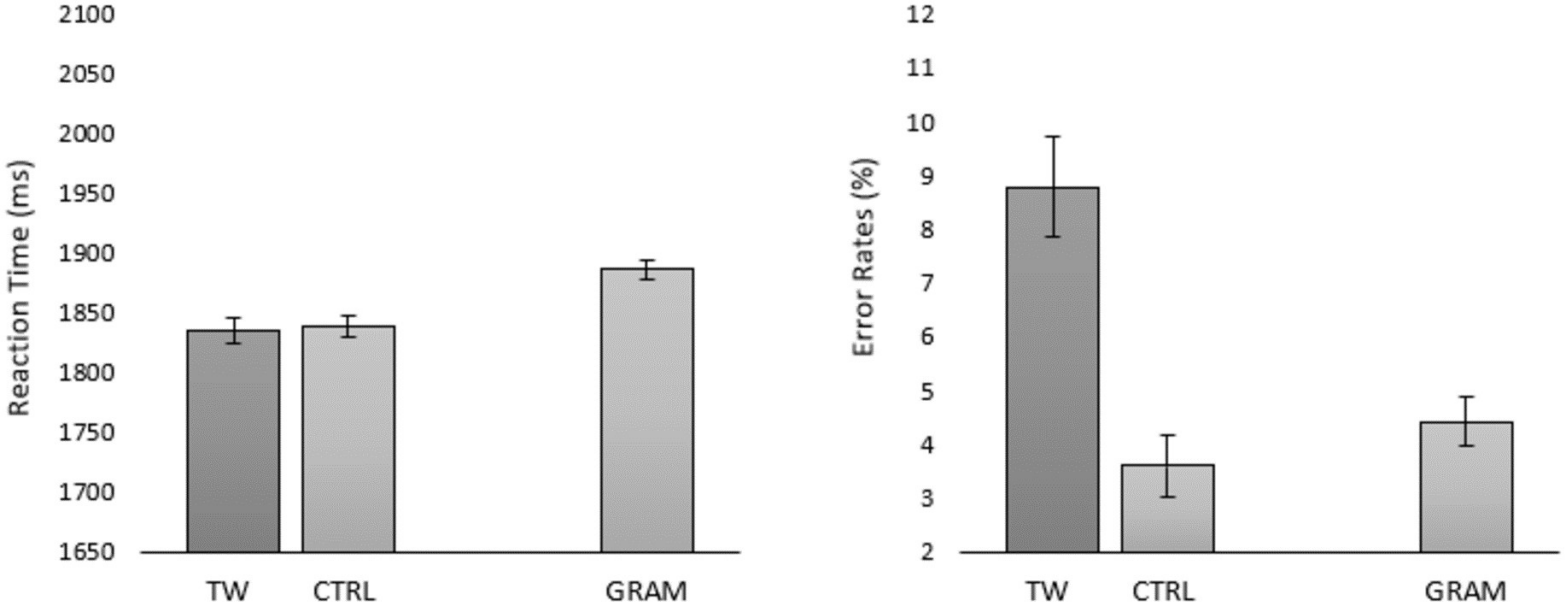
Mean RTs and error rates per condition in Experiment 2. Error bars represent within-participant 95% CIs (Cousineau, 2005). *TW* = transposed-word, *CTRL* = control, *GRAM* = grammatically correct.

Prior to statistical analysis we excluded 6 participants with accuracy lower than 75%. We also excluded trials with RTs < 100 ms and > 3,000 ms (1.61%). The remaining dataset was composed of 14,954 observations. Condition means are shown in Fig 5.

#### Response times

Prior to analysis, we removed incorrect responses (5.26%) and trials with values beyond +/- 2.5 SD from the grand mean (2.86%). The effect of Transposition was not significant (*b* = 0.89; SE = 1.20; *t* = 0.74) with identical mean RTs being obtained for the transposed-word sequences and the control sequences.

#### Error rates

There was a significant effect of Transposition (*b* = 0.72; SE = 0.14; *z* = **4.90**) with more errors in the transposed-word condition compared with the control condition.

#### Conditional accuracy functions

**Fig 6:**
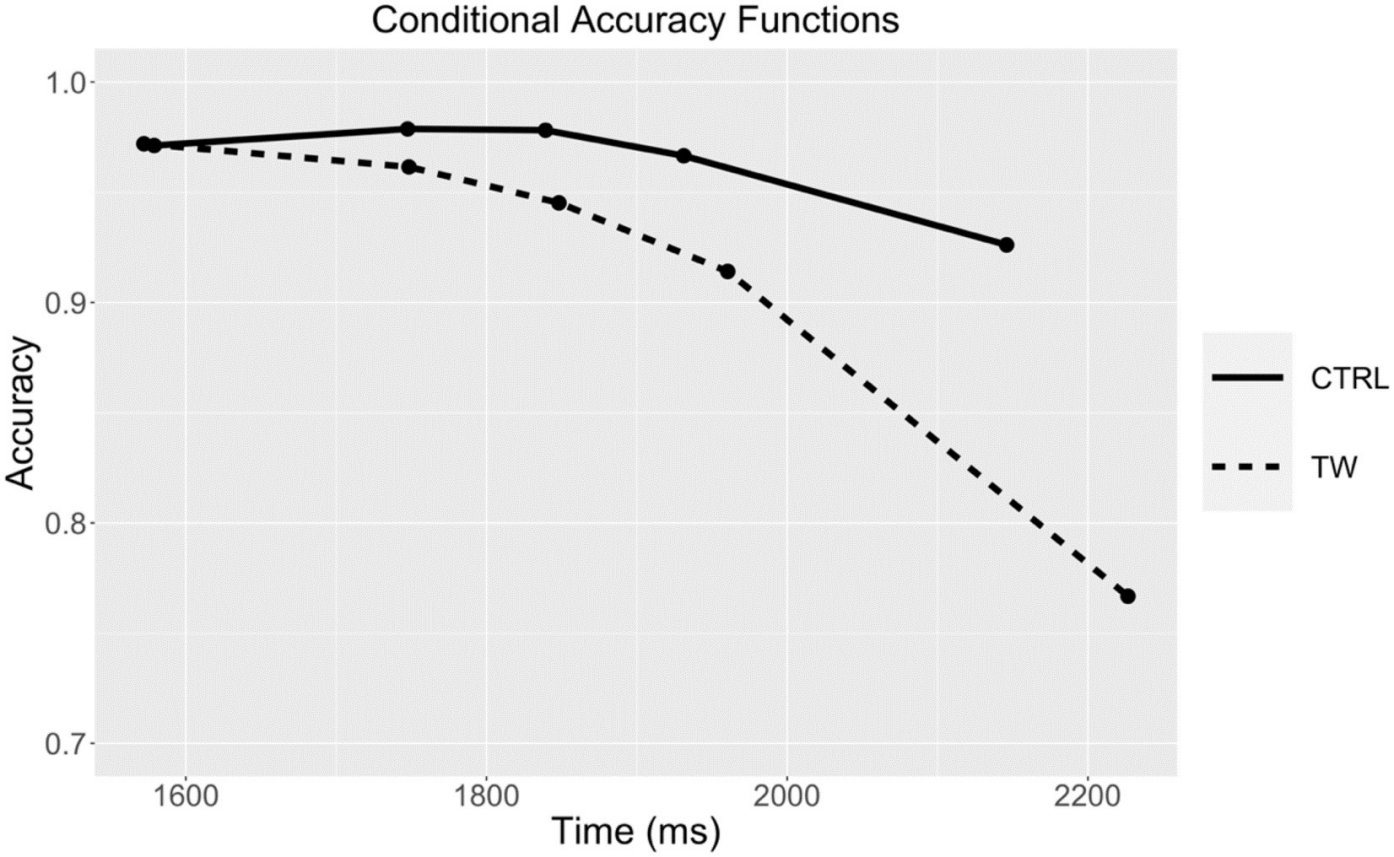
Conditional accuracy functions for the Transposed-word (TW) and Control (CTRL) conditions in Experiment 2. The points represent the accuracy (probability correct) and average RT (in ms) of responses within equal-sized bins (20% of responses per bin).

Fig 6 shows the conditional accuracy functions for the two types of ungrammatical sequence obtained with the RSVP procedure used in Experiment 2. These were calculated in the same way as for Experiment 1.

### Discussion

Experiment 2 provided a replication of Experiment 1, but this time allowing participants to respond during the RSVP sequence (and stopping presentation of the sequence upon participants’ response). In Experiment 2 we found the exact same pattern of effects as in Experiment 1—a transposed-word effect in error rates and no effect on RTs. Moreover, the conditional accuracy functions again revealed that the effects in error rates increased as average RT increased.

In Experiment 3 we provide a direct comparison of transposed-word effects obtained with parallel presentation of words and with the RSVP presentation conditions tested in Experiment 2. Prior work investigating transposed-word effects under serial and parallel presentation conditions (Liu et al. [17]) used RSVP with 250 ms per word and compared that to parallel presentation of words until participants’ response (see also Huang & Staub [13], and Milledge et al. [14]). Crucially, compared to these studies, Experiment 3 enabled a comparison of transposed-word effects under parallel and serial presentation conditions matched for the total presentation duration of word sequences.

## Experiment 3

### Methods

#### Participants

Two hundred participants (100 female) participated in the experiment. They were recruited online via the Prolific platform (Palan & Schitter [19]). They reported having normal or corrected-to-normal vision and ranged in age from 18 to 65 years (M = 29.89 years, SD = 9.41). There were all native French speakers and received monetary compensation (£9/hour). They were naïve to the purpose of the experiment and signed online an informed consent form before starting the experiment.

#### Design and stimuli

The stimuli were the same as in the previous experiments. The design was the same as in the previous experiments except for the addition of the Procedure factor (parallel vs. serial). Each participant was tested with both procedures, with half of the participants receiving the parallel presentation trials first and the other half the serial presentation trials first. The design was therefore a 2 (Transposition: Transposed vs. Control) x 2 (Procedure: parallel vs. serial) factorial. The same stimuli were tested with the two procedures, but target repetition was avoided by using the two counterbalanced lists of stimuli of Experiments 1 and 2. So, participants were tested with both lists (as opposed to one list in the previous experiments), one with parallel presentation and one with serial presentation.

#### Procedures

**Fig 7:**
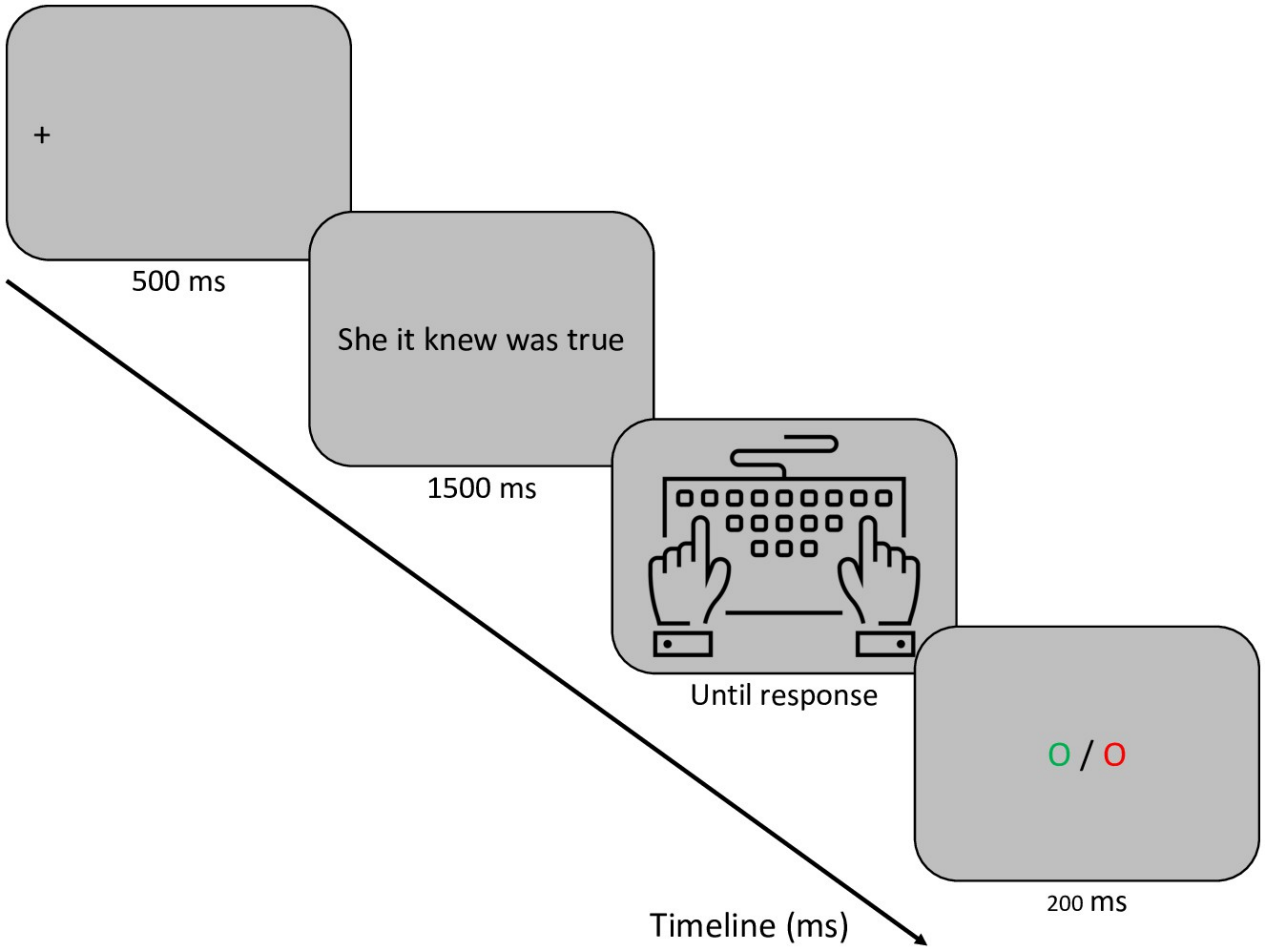
Schematic of one experimental trial for the parallel presentation procedure of Experiment 3. As in the previous experiments, responses were given via the left and right arrow keys of the keyboard.

*Parallel presentation*. A description of one experimental trial is shown in Fig 7. On each trial, a fixation cross was displayed on the left edge of the screen (400 pixels away from the center) during a random time ranging between 300 and 500 ms, followed by the 5-word sequence centered on the screen for 1500 ms. Participants were instructed to decide as rapidly and as accurately as possible whether the sequence of words was grammatically correct or not and could respond before stimulus offset. After participants’ response, a feedback dot was presented for 200 ms, in green if the response was correct or in red if the response was incorrect.

*Serial presentation (RSVP)*. The procedure was the same as for Experiment 2 (i.e., participants could respond before the end of the RSVP stream).

#### Analyses

The same as in the previous experiments, except for the addition of the Procedure factor in the analyses, and the interaction term between Transposition and Procedure. We also added Delta plots for the effects of Transposition in RTs. Correct RTs for all participants were first divided into equal-sized bins (quantiles) separately for the transposed-word and control conditions and for each procedure. The transposed-word effect (mean RT for the transposed-word condition minus mean RT for the control condition) was then calculated for each bin. Delta plots reveal how effects of a variable (here the effects of Transposition) vary as a function of average RT. These plots provide valuable information given potential differences in average RT across the parallel and serial presentation procedures.

### Results

**Fig 8:**
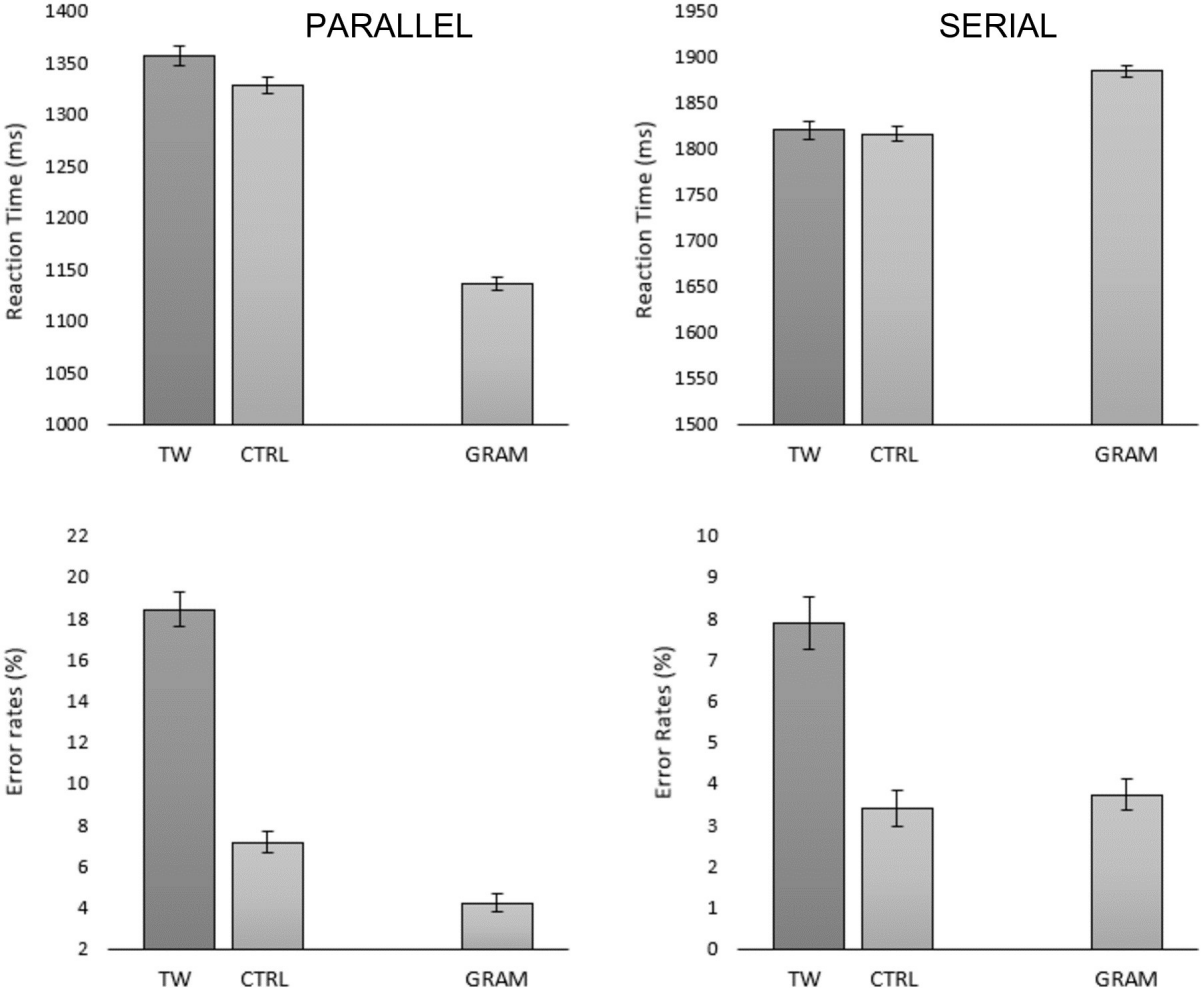
Mean RTs and error rates per condition in Experiment 3 obtained with parallel presentation (left panels) and serial presentation (right panels). Error bars represent within-participant 95% CIs (Cousineau, 2005) [16]. *TW* = transposed-word, *CTRL* = control, *GRAM* = grammatically correct.

Prior to statistical analysis, we excluded 5 participants with accuracy lower than 75%. We also excluded trials with RTs < 100 ms and > 3,000 ms (0.32%). The remaining dataset was composed of 62,200 observations. Condition means are shown in Fig 8.

#### Response times

After removing the trials with incorrect responses (6.36%) we excluded 0.79% of RT values beyond 2.5 SD from the grand mean. We observed a significant effect of Transposition (*b* = 12.83; SE = 1.97; *t* = **6.49**) and of Procedure (*b* = 143.50; SE = 1.44; *t* = **99.13**). Crucially, we found an interaction between Transposition and Procedure (*b* = 12.04; SE = 2.09; *t* = **5.73**) that revealed a greater Transposition effect with parallel presentation (*b* = 12.09; SE = 2.70; *t* = **4.47**) compared to a non-significant effect with serial presentation (*b* = 0.25; SE = 1.12; *t* = 0.22).

#### Error rates

We observed a significant effect of Transposition (*b* = 1.39; SE = 0.06; *z* = **20.52**), a significant effect of Procedure (*b* = 0.95; SE = 0.08; *z* = **11.58**), and a significant interaction between Transposition and Procedure (*b* = 0.21; SE = 0.09; *z* = **2.18**). The interaction reflects the greater effect of Transposition obtained with parallel presentation (*b* = 1.25; SE = 0.05; *z* = **22.13**) compared with serial presentation (*b* = 1.01; SE = 0.08; *z* = **12.66**), although both effects were significant.

#### Conditional accuracy functions and delta plots

**Fig 9:**
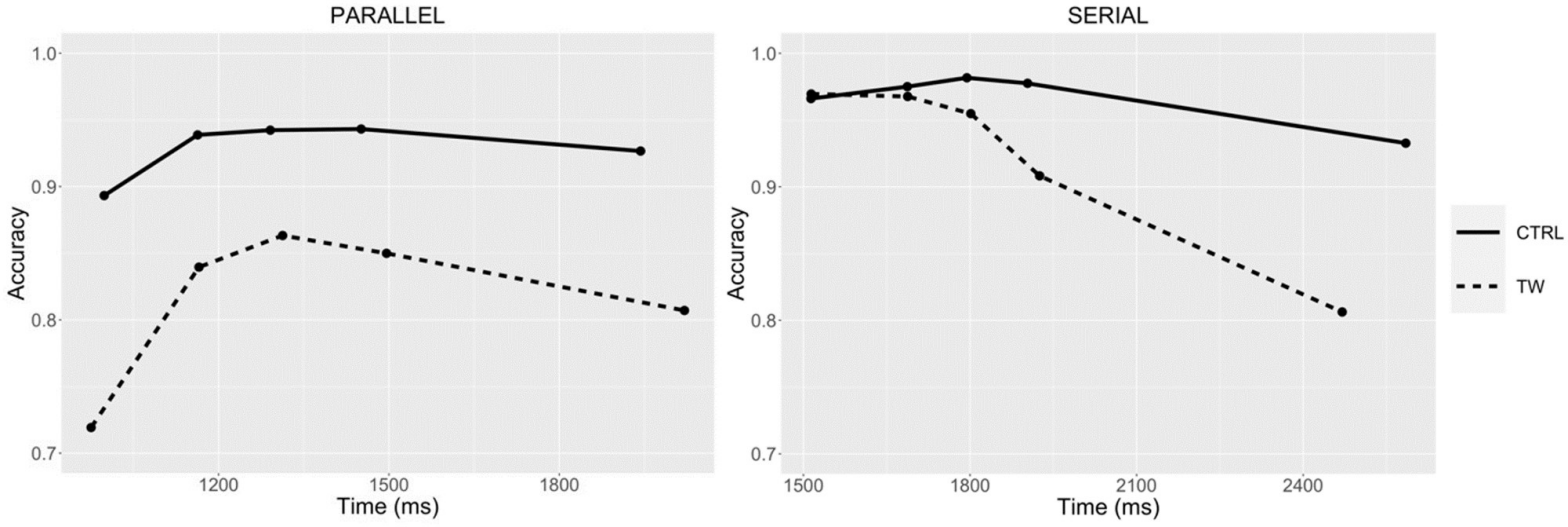
Conditional accuracy functions for the Transposed-word (TW) and Control (CTRL) conditions obtained with parallel presentation (left panel) and serial presentation (right panel) in Experiment 3. The points represent the accuracy (probability correct) and average RT (in ms) of responses within equal-sized bins (20% of responses per bin).

**Fig 10:**
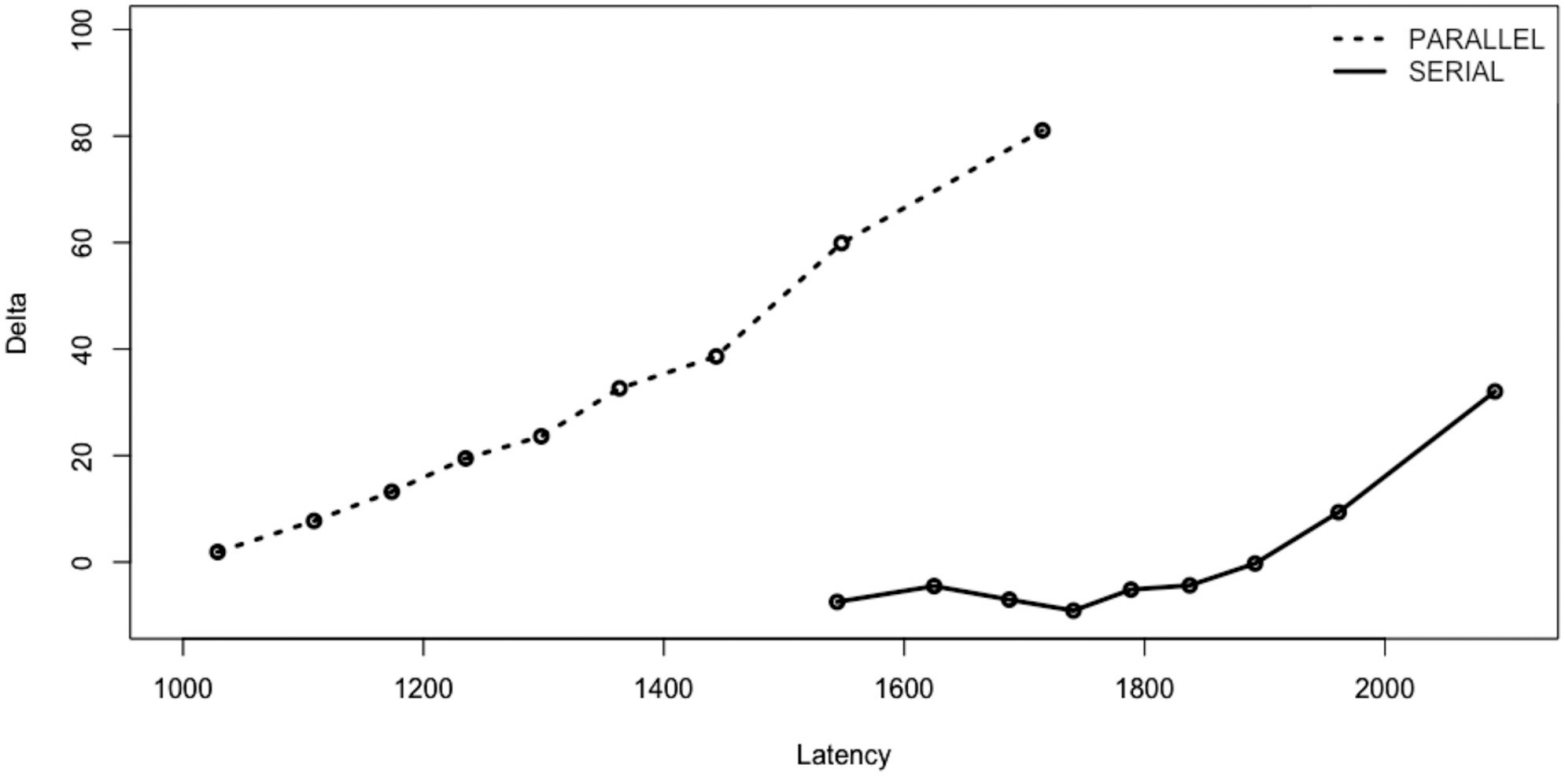
Delta plots of transposed-word effects in RTs (TW—CTRL) in milliseconds obtained with the parallel and serial presentation procedures of Experiment 3. The points represent the average transposition effect observed at the .1, .2, .3, .4, .5, .6, .7, .8, and .9 quantiles calculated separately for each procedure.

The conditional accuracy functions for the two types of ungrammatical sequence and the two procedures tested in Experiment 3 are shown in Fig 9. These were calculated in the same way as for the previous experiments. Fig 10 shows the delta plots for transposed-word effects obtained in the parallel and serial presentation procedures of Experiment 3.

### Discussion

Experiment 3 provided a direct comparison of transposed-word effects obtained under parallel presentation and serial presentation (RSVP) conditions while matching the overall duration of stimuli in the two procedures. We replicated the findings of transposed-word effects obtained in error rates but not in RTs with serial presentation in Experiments 1 and 2, and now crucially demonstrated that the effects emerged in both RTs and error rates with parallel presentation. The conditional accuracy functions again revealed that the effect in error rates under serial presentation increased as RTs increased and showed that this was not the case for parallel presentation. Furthermore, delta plots of the transposition effects in RTs showed that these effects increased as average RT increased. Crucially, the delta plots also revealed that the greater effect obtained with parallel presentation compared with serial presentation was still present when considering the longest RTs in both procedures. Moreover, the delta plots clearly show that it is not just the longer RTs obtained with the RSVP procedure that is the cause of the absence of a transposed-word effect in RTs in this condition, since if anything, the effects should have been stronger with serial presentation.

## General discussion

In three experiments we examined transposed-word effects under conditions where serial reading was imposed by using the RSVP procedure, and Experiment 3 provided a direct comparison of transposed-word effects under serial and parallel presentation conditions. In this critical final Experiment, the total duration of word sequences (1500 ms) was matched across serial and parallel presentation conditions, and participants made grammatical decisions to these sequences. In both procedures, participants could respond prior to stimulus offset.

We found evidence for transposed-word effects in all three experiments, with a strikingly similar pattern seen across the different experiments. That is, participants found it harder to decide that a sequence of words was ungrammatical when that sequence was formed by transposing two words in a regular sentence (e.g., *the white was cat big*) compared with matched ungrammatical sequences that could not be transformed into a correct sentence by transposing any two words (e.g., *the white was cat slowly*). However, contrary to the transposed-word effects observed under normal text presentation conditions (i.e., simultaneous presentation of all words—Mirault et al. [1]), the effects found in the present study were only significant in error rates and not in RTs, except for the parallel presentation conditions tested in Experiment 3. The parallel presentation condition in Experiment 3 replicated the effects seen in both errors and RTs in the Mirault et al. study (see also, Wen et al. [27]). The different pattern of effects seen in Experiment 3 as a function of serial vs. parallel presentation of words replicate the findings of Liu et al. [17] in Chinese.

Do the present findings speak to the more general question concerning the serial vs. parallel processing of words during reading? We predicted a lesser impact of the transposed-word manipulation under conditions of serial reading, principally because serial reading should provide less noisy bottom-up information about word order (e.g., Reichle et al. [28]), hence leading to an overall reduction in transposed-word effects. One could therefore argue that the fact that we did observe this pattern is evidence against a strictly serial, one word-at-time, account of reading. Nevertheless, noisy channel accounts of sentence processing (e.g., Gibson et al. [6]) do predict that errors, including transposed-word errors, can go unnoticed even under strictly incremental processing, due to the approximate (or good-enough: Ferreira & Lowder [9]) nature of the sentence-level structures that are computed on the fly (see also Huang & Staub [13]; Milledge et al. [14]). Moreover, as already proposed in related work from our group (e.g., Dufour et al. [15]; Pegado & Grainger [29]; Wen et al. [27, 30]), top-down constraints imposed by sentence-level structures (syntactic and semantic) would contribute to transposed-word effects independently of presentation mode (the same holds for post-lexical integration accounts of transposed-word effects as proposed by Huang & Staub). Thus, the combination of top-down sentence-level constraints (or post-lexical integration processes) plus noisy bottom-up processing provides a means to capture transposed-word effects under serial reading conditions (for further discussion of the serial vs. parallel reading debate see Huang & Staub [12]; Snell & Grainger [31]).

The key question then is why do the effects only emerge in errors and not in RTs when serial reading is imposed, as in the RSVP conditions tested in the present study? Here we provide a tentative explanation for this pattern, and there are two additional pieces of information that helped forge this explanation. One concerns the fact that correct RTs on grammatical trials were faster than on ungrammatical trials with parallel presentation but slower with serial presentation (see Fig 8). The other concerns the different conditional accuracy functions found with parallel compared with serial presentation (Fig 9). What these two pieces of information suggest is that in the RSVP condition participants were giving their response after seeing the final word in the sequence unless they had already detected an ungrammaticality. This explains the slower RTs on grammatical compared with ungrammatical trials in RSVP. Crucially, since the position in the sequence (from beginning to end) that the ungrammaticality occurred was matched across the transposed-word and control sequences (see Table 1), these early “ungrammatical” responses are largely insensitive to the transposition manipulation. This explains why most of the transposed-word effects seen in error rates in RSVP occurred with the longest RTs (see Fig 9). This also accounts for why there are no transposed-word effects in RTs under serial presentation (RSVP). The slowest RTs, that would otherwise be the main driving force behind transposed-word effects (see the Delta plots in Fig 10), are transformed into errors simply because participants are respecting instructions to respond as rapidly as possible. In other words, participants were trading-off speed for accuracy with the longest RTs and given insufficient evidence to decide that the word sequence is ungrammatical, then the default response is “grammatical”.

A related issue that needs to be addressed is why are transposed-word effects also seen in RTs as well as error rates under parallel presentation, and more generally, why are the effects greater with parallel presentation? Fig 8 reveals that responses to correct sentences were faster and more accurate with parallel presentation. On the other hand, although RTs to ungrammatical sequences were also faster with parallel presentation, there were roughly twice as many errors compared with serial presentation. We would tentatively suggest that with parallel presentation, participants are gradually accumulating evidence in order to make a grammatical decision, and this evidence accumulation receives input from several words at a time (i.e., partly parallel processing of words), and begins as soon as the stimulus appears. This allows participants to set a low criterion for a “grammatical” response while respecting instructions to respond as rapidly and as accurately as possible. Since the criterion for an “ungrammatical” response is tied to the value set for a “grammatical” response (cf., the Leaky Competing Accumulator and Drift Diffusion models of lexical decision: Dufau et al. [32]; Ratcliff et al. [33]; see also Perea et al. [34]) this encourages fast ungrammatical responses that are associated with an increase in errors across the complete range of RTs, and particularly for transposed-word sequences (see Fig 9). We suspect that the overall greater size of transposed-word effects (in both errors and RTs) seen with parallel presentation compared with serial presentation is due to a combination of an increase in bottom-up positional noise under parallel presentation (see also Huang & Staub [13]), plus the different ways that participants perform the grammatical decision task with these two procedures.

In sum, we examined transposed-word effects in conditions that imposed a one-word-at-a-time serial reading by using the RSVP procedure. We found significant transposed-word effects in all experiments, but only in error rates and not in RTs with serial presentation of words. Parallel presentation of words, on the other hand, revealed effects in both RTs and error rates. We conclude that serial presentation reduces uncertainty in the bottom-up process of associating word identities with their positions in a sequence of words and modifies the way in which participants perform the grammatical decision task. Moreover, even with reduced noise in bottom-up processing, top-down sentence-level constraints (and/or post-lexical integration processes) continue to drive transposed-effects independently of how words are presented.
